# Contrast-enhanced endoscopic ultrasound for differential diagnosis of pancreatic cancer: an updated meta-analysis

**DOI:** 10.18632/oncotarget.18915

**Published:** 2017-07-01

**Authors:** Xing-Kang He, Yue Ding, Lei-Min Sun

**Affiliations:** ^1^ Department of Gastroenterology, Sir Run Run Shaw Hospital, Zhejiang University Medical School, Hangzhou 310016, China; ^2^ Institute of Gastroenterology, Zhejiang University, Hangzhou 310016, China

**Keywords:** contrast-enhanced EUS, contrast-enhanced doppler EUS, contrast-enhanced harmonic EUS, pancreatic adenocarcinomas

## Abstract

**Aim:**

We aim to assess the diagnostic value of contrast-enhanced endoscopic ultrasound (CE-EUS) for pancreatic cancer and inflammatory lesions by pooling current evidence.

**Materials and Methods:**

A systematical search of PubMed, Web of Science and the Cochrane Library was performed from inception to January 2016. Two authors independently screened and extracted detailed data from included studies. A random effect model was adopted to estimate the pooled sensitivity, specificity in order to determine the diagnostic ablitity of CE-EUS. Furthermore, we conducted the meta-regression and subgroup analyses to explore possible heterogeneity.

**Results:**

Eighteen eligible studies enrolling 1668 patients were finally included in the study. The pooled sensitivity of CE-EUS for distinguishing pancreatic cancers from solid inflammatory masses was 0.93 (95% CI, 0.91–0.94), and the specificity was 0.88 (95% CI, 0.84–0.90). The area under summary receiver operating characteristic curve yielded 0.97. No publication bias was observed by Deeks’ funnel plot in current meta-analysis.

**Conclusions:**

We provided evidence that CE-EUS is a promising modality for differential diagnosis of pancreatic adenocarcinomas. Further multicenter prospective studies should be carried out to certify its utility.

## INTRODUCTION

Endoscopic ultrasonography (EUS) is considered as a valuable diagnostic technology for pancreatic diseases with good spatial resolution [1, 2]. Although the sensitivity of EUS is high, its ability to characterize and differentiate solid masses is still limited [3]. However, it is crucial for clinicians to establish or exclude pancreatic malignancy in clinical works. Distinguishing pancreatic cancer from inflammatory lesions remains challenging with conventional EUS. The development of EUS-guided fine needle aspiration (EUS-FNA) makes it possible for characterization of pancreatic lesions with high accuracy [4–6]. EUS-FNA is effective in differentiation of pancreatic masses. However, EUS-FNA had its own limitations, including sampling errors and invasive procedure [7, 8]. In addition, the sensitivity of EUS-FNA significantly reduced to 54%–73% with the setting of chronic pancreatitis [9, 10]. It is still imperative for endoscopists to seek for effective and noninvasive technologies that could differentiate pancreatic cancer accurately.

Contrast-enhanced ultrasonography was used in percutaneously abdominal ultrasound examination since 1995, Contrast-enhanced endoscopic ultrasonography (CE-EUS) is also been performed to determine the pancreatic parenchymal perfusion and microvessels inside lesions of interest with better delineation [11]. The application of contrast agents in EUS had improved characterization ability of pancreatic masses and aided in the differentiation of pancreatic diseases. Several studies had evaluated the diagnostic ability of CH-EUS for pancreatic lesions (sensitivity, 80%–100%, specificity, 64%–100%) [12–28]. In 2012, results from a meta-analysis with limited population showed that pooled sensitivity and specificity of CE-EUS were 94% and 89%, respectively [29]. Recently, with the advent of second-generation contrast agents and quantitative analyses [26, 28], a large amount of trials assessed pancreatic solid masses using CE-EUS [16, 19, 20, 23, 26]. Given this background, we perform an updated meta-analysis based on current lectures to assess diagnostic value of CE-EUS for characterization and differentiation pancreatic lesions.

## RESULTS

### Characteristics and quality assessment of included study

**Figure 1:**
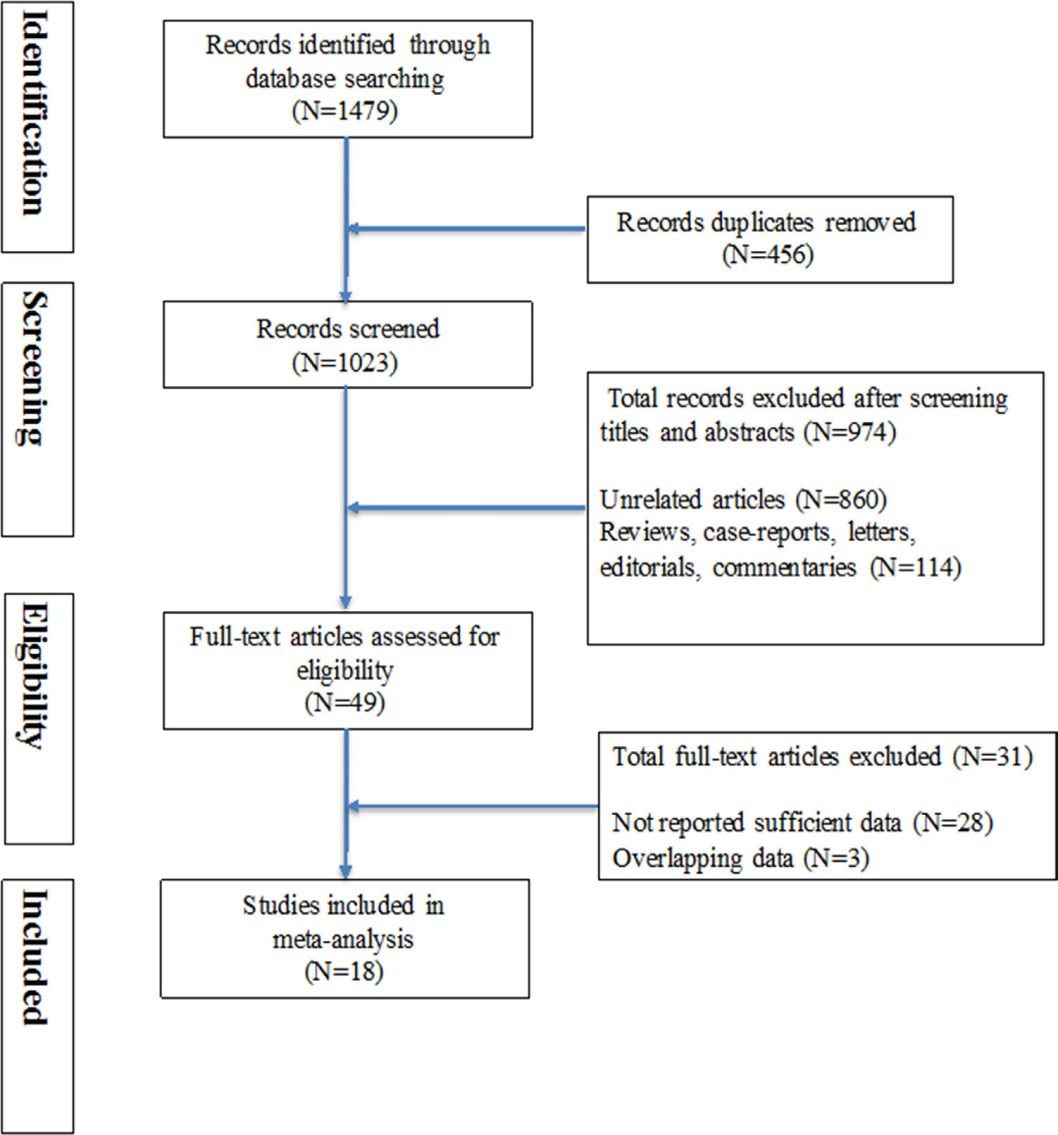
Flow diagram of the literature selection procedure

**Figure 2:**
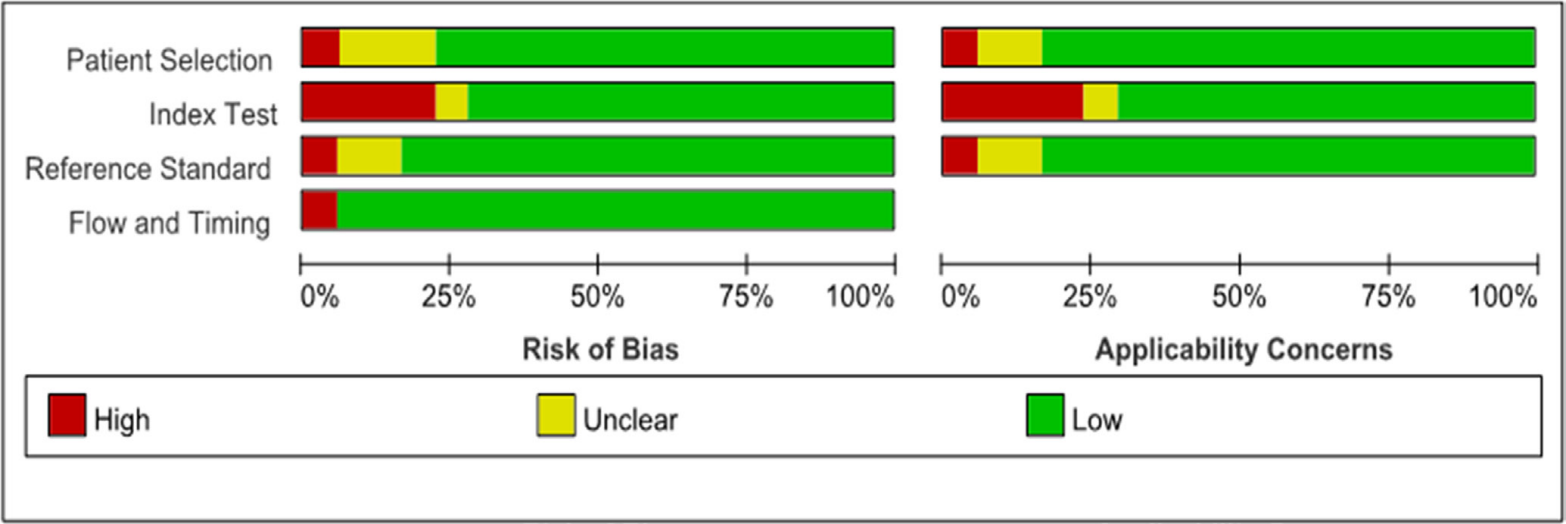
Quality assessment of included studies according to the quality assessment of diagnostic accuracy studies criteria-2 Red color indicated high risk of bias, Yellow color indicated unclear risk of bias, Green color indicated low risk of bias.

Our initial search identified 1475 articles from databases. After applied inclusion and exclusion criteria, 18 eligible studies comprising 1668 participants were included in final analysis. Detailed selection flow was presented in Figure 1, The main characteristics are presented in Table 1. Ten studies were performed in Europe, seven were conducted in Asia and one was in USA. The gold diagnostic standard was based on pathology histology, or follow-up. According to QUADAS-2 criteria, the overall methodological quality of included articles was moderate to high (Figure 2).

**Table 1:** Characteristics of the selected studies

Study	Country	No. of patients	Sex (M/F)	Age (mean, y)	Diagnostic standard	Contrast agent	Contrast mode	Gold standard
Becker et al. 2001 [12]	Germany	23	16/7	58.3	Hypoenhancement	Optison	Color/power Doppler	Histology, follow-up (6 m)
Hocke et al. 2008 [17]	Germany	194	119/75	64	Irregular arterial vessels, no venous vessels	Sonovue	Power Doppler	Histology,
Dietrich et al. 2008 [13]	Germany	93	Unclear	Unclear	Hypoenhancement	Levovist	Color Doppler	Histology,
Sakamoto et al. 2008 [27]	Japan	156	Unclear	Unclear	Hypoenhancement	Levovist	Power Doppler	Histology
Saftoiu et al. 2010 [25]	Romania	54	43/11	56.9	Contrast-enhanced PDVI cut-off < 20%	Sonovue	Power Doppler	Histology, follow-up (> 6 m)
Seicean et al. 2010 [28]	Romania	30	25/5	57	Cut-off < 0.17	Sonovue	Harmonic	Histology, follow-up (9 m)
Napoleon et al. 2010 [22]	France	35	19/16	60	Hypoenhancement	Sonovue	Harmonic	Histology, follow-up (> 12 m)
Fusaroli et al. 2010 [14]	Italy	90	44/46	67	Inhomogeneous hypoenhancement	Sonovue	Harmonic	Histology, follow-up (> 12 m)
Matsubara et al. 2011 [21]	Japan	91	61/30	61.4	Hypoenhancement	Sonazoid	Harmonic	Histology, follow-up (> 12 m)
Romagnuoloet al. 2011 [24]	USA	21	Unclear	Unclear	Hypoperfusion or perfusion defects	Definity	Harmonic	Histology, follow-up (6 m)
Kitano et al. 2011 [19]	Japan	277	173/104	64.3	Hypoenhancement	Sonazoid	Harmonic	Histology, follow-up (> 12 m)
Imazu et al. 2012	Japan	30	22/8	66.9	maximum intensity gain cut-off < 12.5	Sonazoid	Harmonic	Histology, follow-up (> 12 m)
Lee et al. 2013 [20]	Korea	37	24/13	62.3	Hypoenhancement	Sonovue	Harmonic	Histology,
Gheonea et al. 2013 [15]	Roumania	51	25/26	Unclear	Time intensity curve analysis	Sonovue	Harmonic	Histology, follow-up (6 m)
Gincul et al. 2014 [16]	France	100	51/49	64.6	Hypoenhancement	Sonovue	Harmonic	Histology, follow-up (12 m)
Park et al. 2014 [23]	Korea	90	62/28	63.5	Hypoenhancement	Sonovue	Harmonic	Histology, follow-up
Saftoiu et al. 2015 [26]	Multicenter (Romania, Denmark, Germany, Spain).	167	127/40	62	Hypoenhancement	Sonovue	Harmonic	Histology, follow-up (6 m)
Yamashita et al. 2015	Japan	147	92/55	69	hypovascular pattern and lower intensity of enhancement	Sonazoid	Harmonic	Histology

Abbreviation: M/F, male/female; PDVI, Power Doppler Vascularity Index.

### Diagnostic value of contrast-enhanced EUS

**Figure 3:**
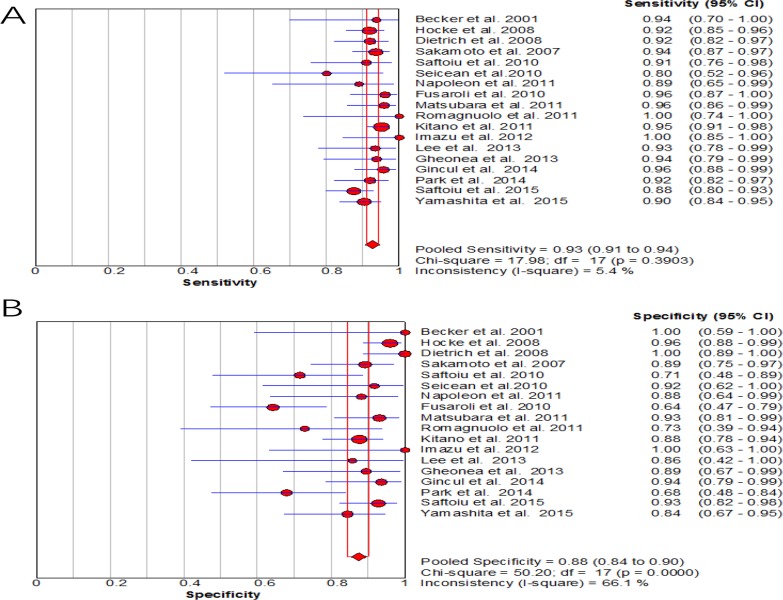
Forest plot of pooled sensitivity and specificity for diagnostic value of CE-EUS (**A**) Sensitivity; (**B**) Specificity. Low heterogeneity across pooled sensitivity (*I*^2^ < 30%) and High heterogeneity across pooled specificity (*I*^2^ > 50%).

**Figure 4:**
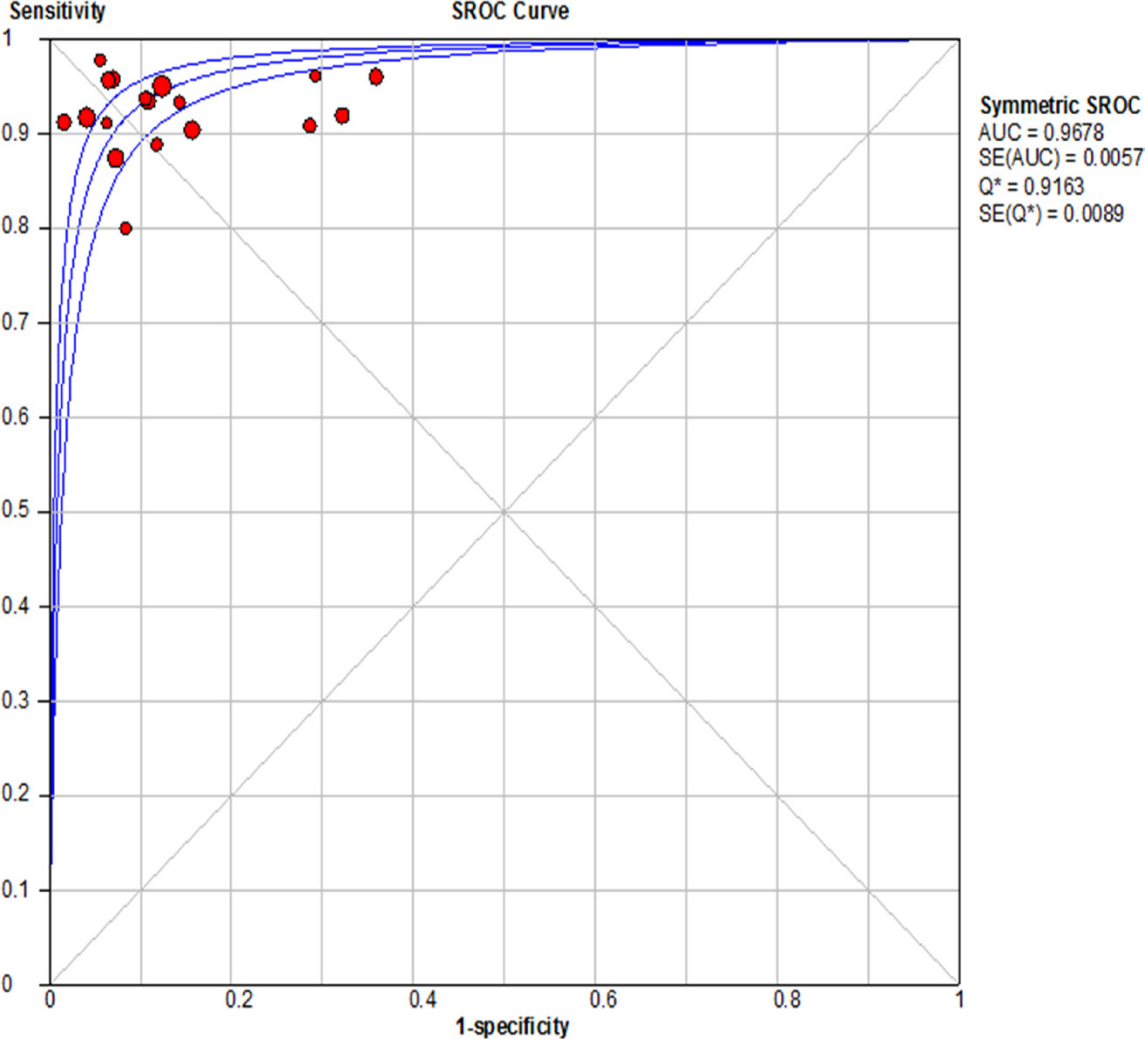
Summary receiver operating characteristic (SROC) curve for the diagnostic accuracy of CE-EUS AUC (Area Under Curve) of 0.97 indicated a a perfect test. SE, standard error.

**Figure 5:**
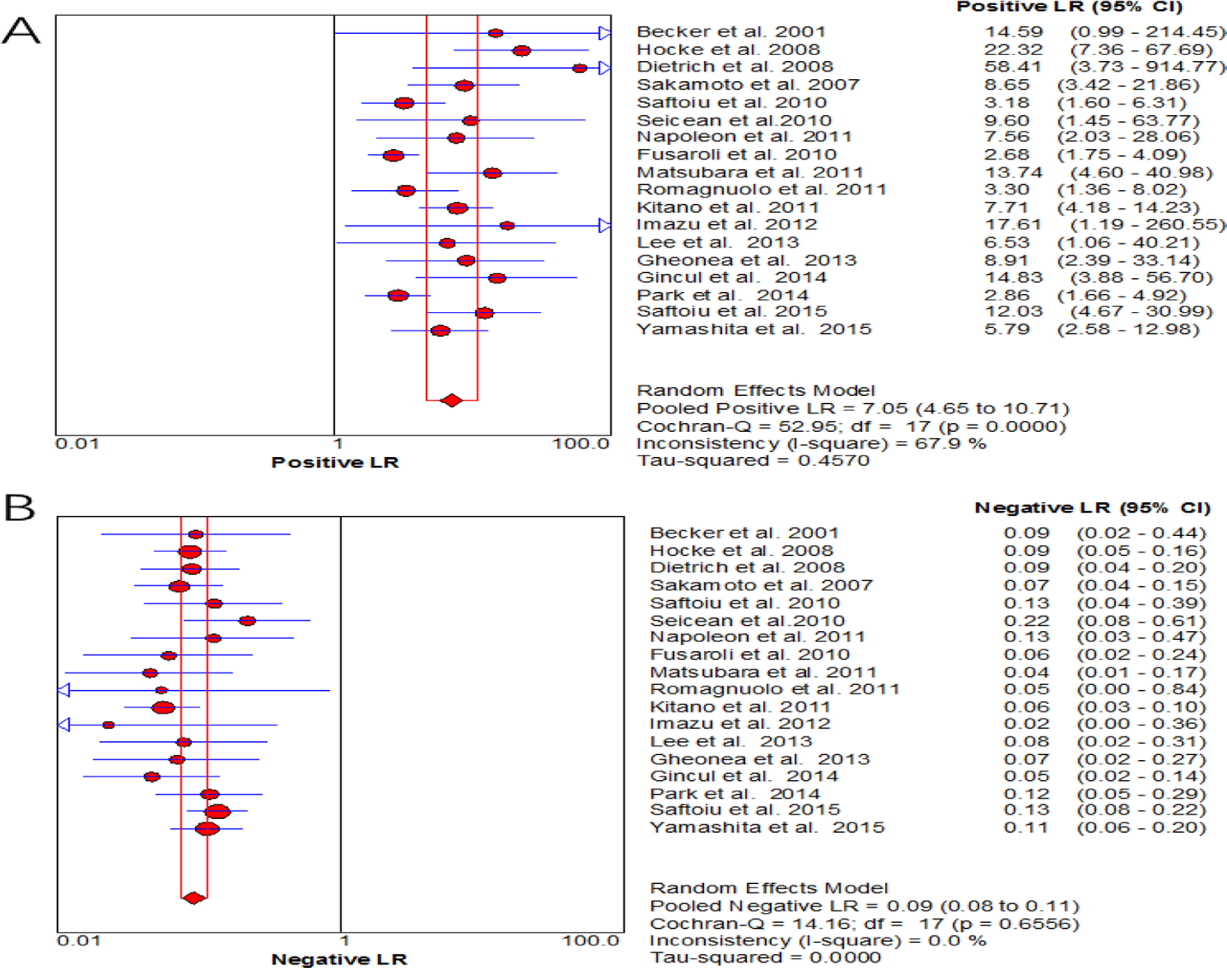
Forest plot of positive likelihood ratio and negative likelihood ratio for CE-EUS (**A**) forest plots of the positive likelihood ratio; (**B**) forest plots of negative likelihood ratio. High heterogeneity across pooled positive likelihood ratio (*I*^2^ > 50%)and Low heterogeneity across pooled negative likelihood ratio (*I*^2^ < 30%).

**Figure 6:**
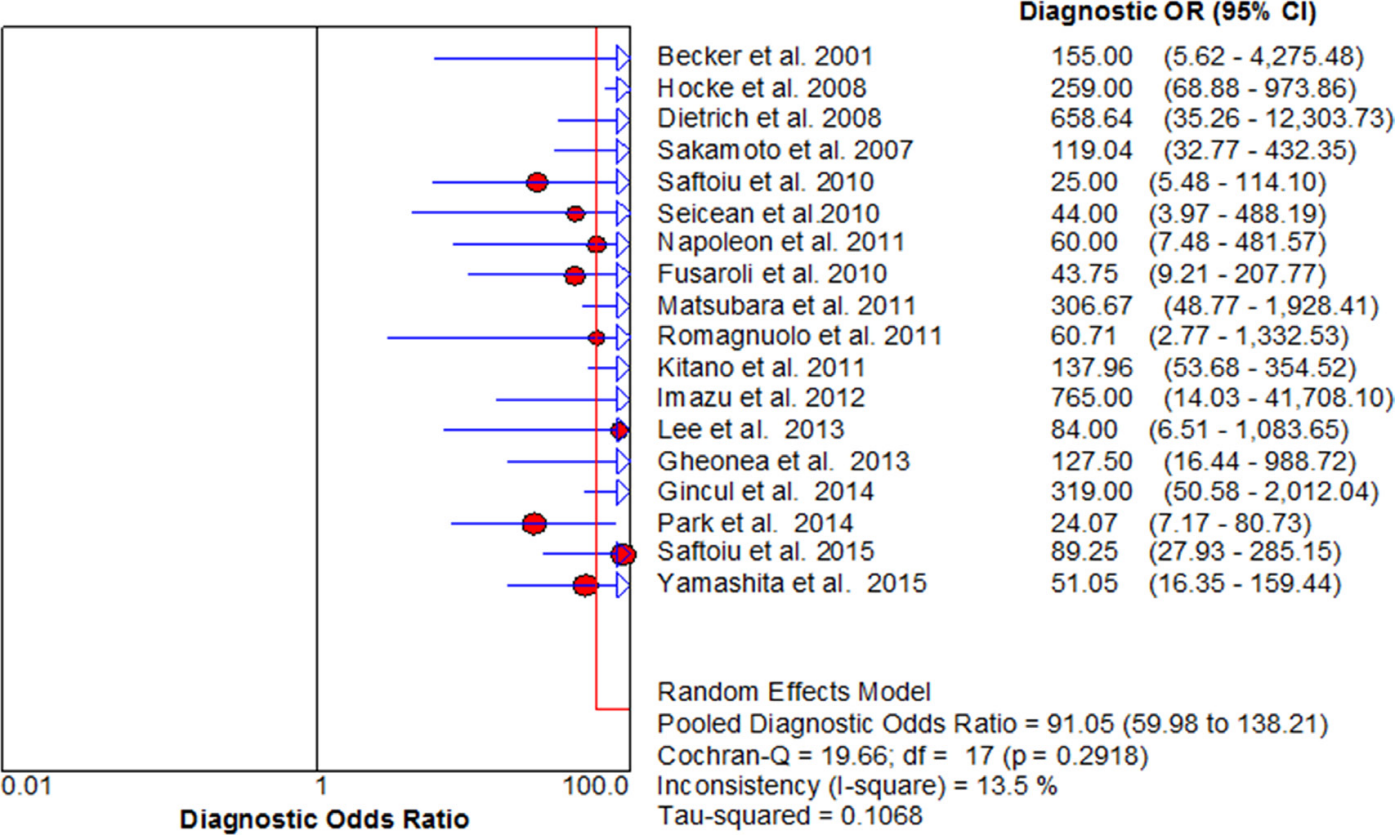
Forest diagnostic odds ratio of CE-EUS Low heterogeneity across pooled diagnostic odds ratio (*I*^2^ < 30%).

**Figure 7:**
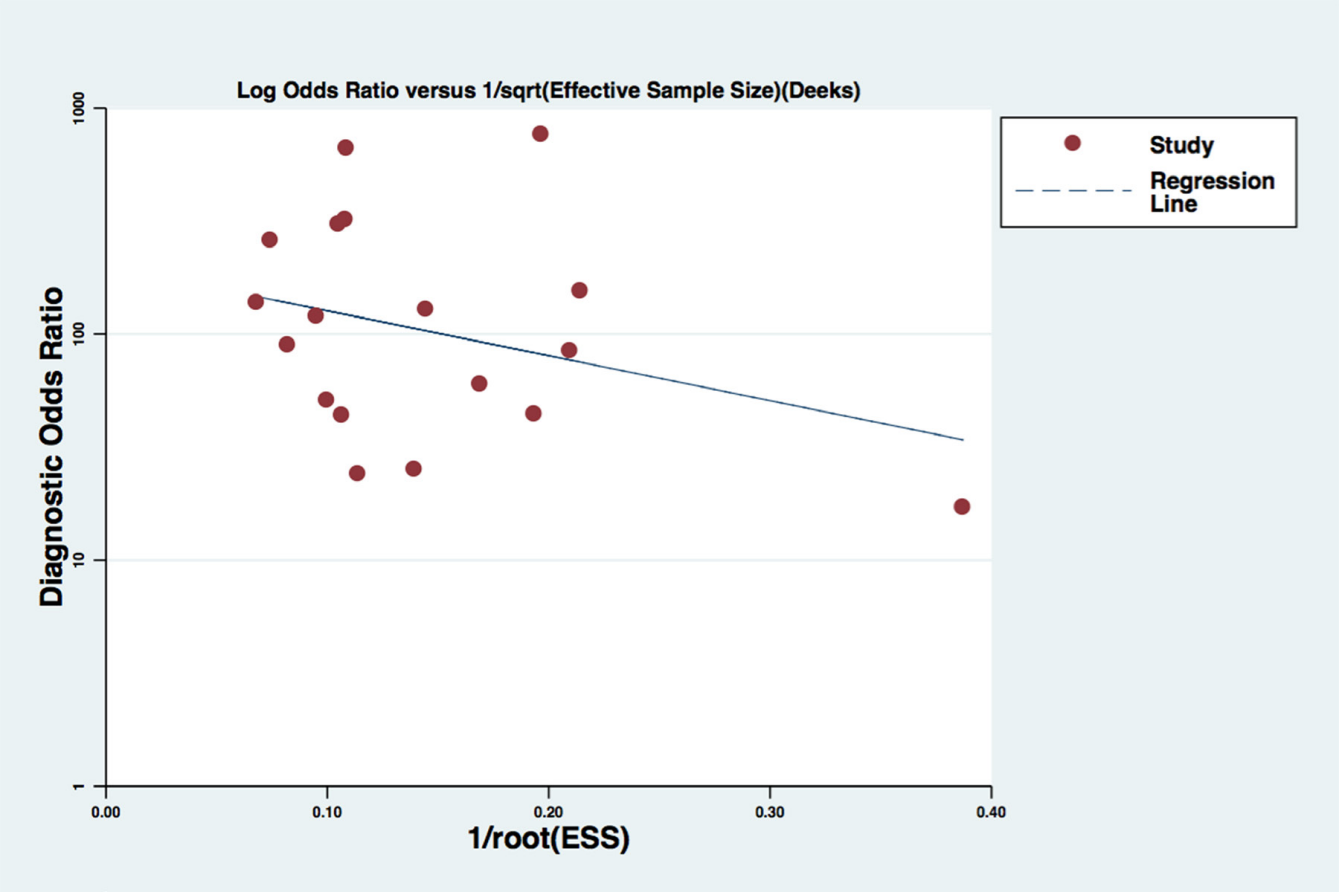
Publication bias of selected studies by Deeks’ funnel plot

For CE-EUS, the pooled estimates of sensitivity and specificity were 93% (95% CI, 0.91–0.94) and 88% (95% CI, 0.84–0.90), respectively (Figure 3). There was no significant heterogeneity in sensitivity (*P =* 0.39, *I*^2^ = 5.4%), while significant heterogeneity was observed in specificity (*P <* 0.001, *I*^2^ = 66.1%) (Figure 3). The area under the SROC was 0.97 (Figure 4). The pooled positive and negative likelihood ratios were 7.05 (95% CI, 4.65–10.71) and 0.09 (95% CI, 0.08–0.11) in diagnosis of pancreatic cancer (Figure 5). Diagnostic odds ratio was 91.05 (95% CI, 59.98–138.21), indicating a high value of diagnostic efficacy of CE-EUS (Figure 6). No significant risk of publication bias was observed in our study for CE-EUS by Deeks’ funnel plot (*P =* 0.967) (Figure 7).

### Meta-regression and sensitivity analysis

To explore possible heterogeneity, we performed a meta-regression analysis and results showed that the characteristics of studies were not significantly associated with diagnostic odds ratio (Table 2). It is illustrated by Figures 3–6 that two studies of Park et al. 2014 and Fusaroli et al. 2010 were outliers. After exclusion of them, sensitivity analysis still demonstrated the consistence of main results (Table 3).

**Table 2:** Meta-regression for the potential source of heterogeneity

Study characteristic	Relative Diagnostic odd ratio (95% CI)	*P* value
patient (< 60 patients vs. ≥ 60 patients)	1.56 (0.56, 4.33)	0.37
contrast mode (color/power Doppler vs. harmonic)	0.92 (0.33, 2.58)	0.87
country (Europe vs. other)	0.87 (0.34, 2.21)	0.76
analysis of images (quality vs. quantity)	0.93 (0.30, 2.92)	0.89

CI, Confidence interval.

**Table 3:** Subgroup analysis by exclusion of outliers

The pooled results	Pooled value (95% CI)	*P* value	I^2^ (%)
Sensitivity	0.93 (0.91, 0.94)	0.33	11.2
Specificity	0.91 (0.88, 0.93)	0.06	37.6
Positive likelihood ratio	8.10 (5.74, 11.42)	0.12	31
Negative likelihood ratio	0.09 (0.07, 0.11)	0.57	0
Diagnostic OR	110.44 (73.42, 166.11)	0.58	0

CI, Confidence interval; *I*^*2*^, inconsistency; *I*^*2*^ > 50% was considered significant for heterogeneity.

## DISCUSSION

It is well known that pancreatic cancer is a lethal disease with dismal prognosis duo to low eraly detection rate. The 5-year survival rate was sharply decreased by advanced stage [30]. Patients were free of symptoms until they were diagnosed by advanced pancreatic cancer [31]. There is an imperative need to diagnose pancreatic cancer at earlier stages. Regarding accurate differentiation of pancreatic cancers from inflammatory tumor-like lesions still remains a big challenge for clinicians and is crucial for therapeutic decisions [32].

It is well demonstrated by several studies [33–35] that EUS is super to other modalities in detection and diagnosis of pancreatic diseases with high sensitivity. However, the ability to characterize solid lesions accurately still remains limited, particularly in the setting of chronic pancreatitis.

CE-EUS, with the intravenously infusion of contrast agents, is a newly developed technology. It can characterize and differentiate pancreatic lesions non-invasively [11]. In general, CE-EUS could be classified as contrast-enhanced Doppler EUS (CD-EUS) and contrast-enhanced harmonic EUS (CH-EUS) according to the method of sonographic assessment [36]. For CD-EUS, intravenous contrast agents would enhance the Doppler signals from vascularity of targeted lesions [37, 38]. However, the disadvantage of this technique included the flash and blooming artifacts. Furthermore, the poor ability to depict microvessels with slow flow and parenchymal perfusion also limited its application widely [27, 39]. For these limitations, CH-EUS was developed to overcome them. It depicts harmonic signals from contrast agents selectively and filters them from surrounding tissues [40, 41]. Thereby, it provides more detailed images of fine vessels with slow flow and parenchymal perfusion in the target lesions [42, 43]. This allows pancreatic lesions to be visualized and characterized more accurately. Besides, the second-generation of contrast agents also demonstrated to be relatively safe for patients, even with liver and renal dysfunctions [43–46].

In our meta-analysis, the pooled results supported a great diagnostic value of CE-EUS for characterization and differentiation of pancreatic masses, which were consistent with previous meta-analysis and studies [29]. Compared with previous meta-analysis, the strength of our study is included comprehensive lectures. CE-EUS is extremely useful for patients with negative results of EUS-FNA. Although EUS-FNA still is considered as a gold standard for pancreatic cancer diagnosis, the sensitivity and accuracy is still suboptimal, particularly in the setting of chronic pancreatitis [47]. Several trials reported that CH-EUS could complements EUS-FNA by delineating the outline of the target lesions clearly, thus facilitating EUS-FNA [14, 19, 48]. Moreover, it could not only improve the sensitivity of EUS-FNA, but also avoid repeated biopsy or surgery. For CH-EUS, a well-known and sensitive diagnostic standard of pancreatic adenocarcinomas is a hypoenhanced image of lesion [19, 22]. age olesion [19, 22]. However, it is somehow operator-dependent and subjective to the analysis of the enhanced pattern, which might affect the diagnostic accuracy. To avoid this disadvantage, a quantification analysis, time–intensity curve for region of interest, was developed recently [15, 18, 21, 28]. Five of the included studies demonstrated the values of maximum intensity, median intensity, time to peak, intensity reduction rate, the ratio of uptake inside the mass to uptake of the surrounding parenchyma in discrimination of malignances from pancreatitis, solid-pseudopapilliary neoplasm and neuroendocrine tumors [15, 18, 21, 26, 28], which makes objective definition of lesion characteristics possible. CH-EUS is also a reproducible method in the evaluation of pancreatic lesions with good interobserver agreement, even for endosonographers with no or limited experience in EUS [16].

The present meta-analysis has some limitations. Significant heterogeneity in specificity and positive likelihood ratio might affect interpretation of the data and conclusions. Serval diagnostic criterion for CE-EUS were adopted in included studies, which might introduce some bias into our conclusion. Furthermore, we cannot exclude the presence of publication bias, although the analysis of the funnel plot indicated that it could not be detected.

In conclusion, CE-EUS, especially for CH-EUS, is a promising tool for differential diagnosis of pancreatic cancer. CE-EUS should be regarded as a promising tool for pancreatic masses characterization, especially when EUS-FNA findings were negative. Further multicenter trials should be carried out to certify its utility.

## MATERIALS AND METHODS

### Search strategy

We searched PubMed, Web of Science and the Cochrane Library from inception to January 2016 for relevant articles comprehensively. Following search terms were adopted: (“contrast-enhanced” OR “contrast medium” OR “echo-enhanced”) AND (“pancreatic mass*” OR “pancreatic cancer” OR “pancreatitis” OR “pancreatitis” OR “pancreatic lesion*” OR “pancreatic adenocarcinoma”) AND (“ultrasonograph*” OR “ultrasound” OR “endosonograph*” OR “endosonography” OR “EUS”). We also searched bibliography of articles and reviews to identify additional articles.

### Inclusion and exclusion criteria

Articles were considered as eligible if they used CE-EUS for the diagnosis, provision of data for true positive (TP), false positive (FP), false negative and true-negative (TN), the reference standard based on histopathology of samples by EUS-FNA, surgery or a follow-up of at least 6 months. Following studies were excluded: (1) complete data unavailable; (2) overlapping with the selected articles; (3) Case reports, reviews, editorials, comments, abstracts.

### Data extraction

Two authors independently extracted following data from each study: authors, year, country, numbers of patient, sex, age, diagnostic standard, contrast agent, contrast mode, gold standard. We adopted the Quality Assessment of Diagnostic Accuracy Studies-2 (QUADAS-2) for quality assessment [49]. Any discrepancies were resolved by discussions or consensus.

### Statistical methods

The pooled estimates of sensitivity, specificity, diagnostic odds ratio were performed by Meta-Disc, version 1.4 (Ramony Cajal Hospital, Madrid, Spain). Heterogeneity across studies was evaluated by the Cochrane *Q* test and I^2^ statistic. We constructed a summary receiver operating characteristic (sROC) curve and calculated the area under sROC (AUC). The meta-regression and sensitivity analyses were performed with the following covariates such as numbers of patient (< 60 patients vs. ≥ 60 patients), contrast mode (color/power Doppler vs. harmonic), country (Europe vs. other), analysis of images (quality vs. quantity). Deeks’ asymmetry test was used to detect publication bias by Stata version 13.0 (Stata Corporation, College Station, Texas). The two-tailed *P* value is statistically significant at less than 0.05.

## Abbreviations

EUS: endoscopic ultrasonography
CE-EUS: contrast-enhanced EUS
CD-EUS: contrast-enhanced Doppler EUS
CH-EUS: contrast-enhanced harmonic EUS
SROC: summary receiver operating characteristic
TP: true positive
FN: false negative
TN: true negative
FP: false positive
AUC: area under the curve
CI: confidence interval
LR: likelihood ratio
QUADAS: quality assessment of diagnostic accuracy studied

## References

[1] Teshima CW, Sandha GS (2014). Endoscopic ultrasound in the diagnosis and treatment of pancreatic disease. World J Gastroenterol.

[2] Rosch T, Lorenz R, Braig C, Feuerbach S, Siewert JR, Schusdziarra V, Classen M (1991). Endoscopic ultrasound in pancreatic tumor diagnosis. Gastrointest Endosc.

[3] Brand B, Pfaff T, Binmoeller KF, Sriram PV, Fritscher-Ravens A, Knofel WT, Jackle S, Soehendra N (2000). Endoscopic ultrasound for differential diagnosis of focal pancreatic lesions, confirmed by surgery. Scand J Gastroenterol.

[4] Hewitt MJ, McPhail MJ, Possamai L, Dhar A, Vlavianos P, Monahan KJ (2012). EUS-guided FNA for diagnosis of solid pancreatic neoplasms: a meta-analysis. Gastrointest Endosc.

[5] Iglesias-Garcia J, Dominguez-Munoz E, Lozano-Leon A, Abdulkader I, Larino-Noia J, Antunez J, Forteza J (2007). Impact of endoscopic ultrasound-guided fine needle biopsy for diagnosis of pancreatic masses. World J Gastroenterol.

[6] Xu C, Li Z, Wallace M (2012). Contrast-enhanced harmonic endoscopic ultrasonography in pancreatic diseases. Diagn Ther Endosc.

[7] Schmidt RL, Witt BL, Matynia AP, Barraza G, Layfield LJ, Adler DG (2013). Rapid on-site evaluation increases endoscopic ultrasound-guided fine-needle aspiration adequacy for pancreatic lesions. Dig Dis Sci.

[8] Matynia AP, Schmidt RL, Barraza G, Layfield LJ, Siddiqui AA, Adler DG (2014). Impact of rapid on-site evaluation on the adequacy of endoscopic-ultrasound guided fine-needle aspiration of solid pancreatic lesions: a systematic review and meta-analysis. J Gastroenterol Hepatol.

[9] Fritscher-Ravens A, Brand L, Knofel WT, Bobrowski C, Topalidis T, Thonke F, de Werth A, Soehendra N (2002). Comparison of endoscopic ultrasound-guided fine needle aspiration for focal pancreatic lesions in patients with normal parenchyma and chronic pancreatitis. Am J Gastroenterol.

[10] Varadarajulu S, Tamhane A, Eloubeidi MA (2005). Yield of EUS-guided FNA of pancreatic masses in the presence or the absence of chronic pancreatitis. Gastrointest Endosc.

[11] Kitano M, Sakamoto H, Matsui U, Ito Y, Maekawa K, von Schrenck T, Kudo M (2008). A novel perfusion imaging technique of the pancreas: contrast-enhanced harmonic EUS (with video). Gastrointest Endosc.

[12] Becker D, Strobel D, Bernatik T, Hahn EG (2001). Echo-enhanced color- and power-Doppler EUS for the discrimination between focal pancreatitis and pancreatic carcinoma. Gastrointest Endosc.

[13] Dietrich CF, Ignee A, Braden B, Barreiros AP, Ott M, Hocke M (2008). Improved differentiation of pancreatic tumors using contrast-enhanced endoscopic ultrasound. Clin Gastroenterol Hepatol.

[14] Fusaroli P, Spada A, Mancino MG, Caletti G (2010). Contrast harmonic echo-endoscopic ultrasound improves accuracy in diagnosis of solid pancreatic masses. Clin Gastroenterol Hepatol.

[15] Gheonea DI, Streba CT, Ciurea T, Saftoiu A (2013). Quantitative low mechanical index contrast-enhanced endoscopic ultrasound for the differential diagnosis of chronic pseudotumoral pancreatitis and pancreatic cancer. BMC Gastroenterol.

[16] Gincul R, Palazzo M, Pujol B, Tubach F, Palazzo L, Lefort C, Fumex F, Lombard A, Ribeiro D, Fabre M, Hervieu V, Labadie M, Ponchon T (2014). Contrast-harmonic endoscopic ultrasound for the diagnosis of pancreatic adenocarcinoma: a prospective multicenter trial. Endoscopy.

[17] Hocke M, Schmidt C, Zimmer B, Topalidis T, Dietrich CF, Stallmach A (2008). Contrast enhanced endosonography for improving differential diagnosis between chronic pancreatitis and pancreatic cancer. [Article in German]. Dtsch Med Wochenschr.

[18] Imazu H, Kanazawa K, Mori N, Ikeda K, Kakutani H, Sumiyama K, Hino S, Ang TL, Omar S, Tajiri H (2012). Novel quantitative perfusion analysis with contrast-enhanced harmonic EUS for differentiation of autoimmune pancreatitis from pancreatic carcinoma. Scand J Gastroenterol.

[19] Kitano M, Kudo M, Yamao K, Takagi T, Sakamoto H, Komaki T, Kamata K, Imai H, Chiba Y, Okada M, Murakami T, Takeyama Y (2012). Characterization of small solid tumors in the pancreas: the value of contrast-enhanced harmonic endoscopic ultrasonography. Am J Gastroenterol.

[20] Lee TY, Cheon YK, Shim CS (2013). Clinical role of contrast-enhanced harmonic endoscopic ultrasound in differentiating solid lesions of the pancreas: a single-center experience in Korea. Gut Liver.

[21] Matsubara H, Itoh A, Kawashima H, Kasugai T, Ohno E, Ishikawa T, Itoh Y, Nakamura Y, Hiramatsu T, Nakamura M, Miyahara R, Ohmiya N, Ishigami M (2011). Dynamic Quantitative Evaluation of Contrast-Enhanced Endoscopic Ultrasonography in the Diagnosis of Pancreatic Diseases. Pancreas.

[22] Napoleon B, Alvarez-Sanchez MV, Gincoul R, Pujol B, Lefort C, Lepilliez V, Labadie M, Souquet JC, Queneau PE, Scoazec JY, Chayvialle JA, Ponchon T (2010). Contrast-enhanced harmonic endoscopic ultrasound in solid lesions of the pancreas: results of a pilot study. Endoscopy.

[23] Park JS, Kim HK, Bang BW, Kim SG, Jeong S, Lee DH (2014). Effectiveness of contrast-enhanced harmonic endoscopic ultrasound for the evaluation of solid pancreatic masses. World J Gastroenterol.

[24] Romagnuolo J, Hoffman B, Vela S, Hawes R, Vignesh S (2011). Accuracy of contrast-enhanced harmonic EUS with a second-generation perflutren lipid microsphere contrast agent (with video). Gastrointest Endosc.

[25] Saftoiu A, Iordache SA, Gheonea DI, Popescu C, Malos A, Gorunescu F, Ciurea T, Iordache A, Popescu GL, Manea CT (2010). Combined contrast-enhanced power Doppler and real-time sonoelastography performed during EUS, used in the differential diagnosis of focal pancreatic masses (with videos). Gastrointest Endosc.

[26] Saftoiu A, Vilmann P, Dietrich CF, Iglesias-Garcia J, Hocke M, Seicean A, Ignee A, Hassan H, Streba CT, Ioncica AM, Gheonea DI, Ciurea T (2015). Quantitative contrast-enhanced harmonic EUS in differential diagnosis of focal pancreatic masses (with videos). Gastrointest Endosc.

[27] Sakamoto H, Kitano M, Suetomi Y, Maekawa K, Takeyama Y, Kudo M (2008). Utility of contrast-enhanced endoscopic ultrasonography for diagnosis of small pancreatic carcinomas. Ultrasound Med Biol.

[28] Seicean A, Badea R, Stan-Iuga R, Mocan T, Gulei I, Pascu O (2010). Quantitative Contrast-Enhanced Harmonic Endoscopic Ultrasonography for the Discrimination of Solid Pancreatic Masses. Ultraschall Med.

[29] Gong TT, Hu DM, Zhu Q (2012). Contrast-enhanced EUS for differential diagnosis of pancreatic mass lesions: a meta-analysis. Gastrointest Endosc.

[30] Bilimoria KY, Bentrem DJ, Ko CY, Ritchey J, Stewart AK, Winchester DP, Talamonti MS (2007). Validation of the 6th edition AJCC Pancreatic Cancer Staging System: report from the National Cancer Database. Cancer.

[31] Siegel R, Ma J, Zou Z, Jemal A (2014). Cancer statistics, 2014. CA Cancer J Clin.

[32] Tamm E, Charnsangavej C (2001). Pancreatic cancer: current concepts in imaging for diagnosis and staging. Cancer J.

[33] Agarwal B, Abu-Hamda E, Molke KL, Correa AM, Ho L (2004). Endoscopic ultrasound-guided fine needle aspiration and multidetector spiral CT in the diagnosis of pancreatic cancer. Am J Gastroenterol.

[34] DeWitt J, Devereaux B, Chriswell M, McGreevy K, Howard T, Imperiale TF, Ciaccia D, Lane KA, Maglinte D, Kopecky K, LeBlanc J, McHenry L, Madura J (2004). Comparison of endoscopic ultrasonography and multidetector computed tomography for detecting and staging pancreatic cancer. Ann Intern Med.

[35] Varadarajulu S, Eloubeidi MA (2010). The role of endoscopic ultrasonography in the evaluation of pancreatico-biliary cancer. Surg Clin North Am.

[36] Jang SI, Lee DK (2014). Contrast-enhanced endoscopic ultrasonography: advance and current status. Ultrasonography.

[37] Kitano M, Sakamoto H, Kudo M (2012). Endoscopic ultrasound: contrast enhancement. Gastrointest Endosc Clin N Am.

[38] Ishikawa T, Itoh A, Kawashima H, Ohno E, Matsubara H, Itoh Y, Nakamura Y, Nakamura M, Miyahara R, Hayashi K, Ishigami M, Katano Y, Ohmiya N (2010). Usefulness of EUS combined with contrast-enhancement in the differential diagnosis of malignant versus benign and preoperative localization of pancreatic endocrine tumors. Gastrointest Endosc.

[39] Kitano M, Kudo M, Maekawa K, Suetomi Y, Sakamoto H, Fukuta N, Nakaoka R, Kawasaki T (2004). Dynamic imaging of pancreatic diseases by contrast enhanced coded phase inversion harmonic ultrasonography. Gut.

[40] Kitano M, Sakamoto H, Komaki T, Kudo M (2011). New techniques and future perspective of EUS for the differential diagnosis of pancreatic malignancies: contrast harmonic imaging. Dig Endosc.

[41] Kitano M, Sakamoto H, Kudo M (2014). Contrast-enhanced endoscopic ultrasound. Dig Endosc.

[42] Hirooka Y, Itoh A, Kawashima H, Ohno E, Itoh Y, Nakamura Y, Hiramatsu T, Sugimoto H, Sumi H, Hayashi D, Ohmiya N, Miyahara R, Nakamura M (2012). Contrast-enhanced endoscopic ultrasonography in digestive diseases. J Gastroenterol.

[43] Reddy NK, Ioncica AM, Saftoiu A, Vilmann P, Bhutani MS (2011). Contrast-enhanced endoscopic ultrasonography. World J Gastroenterol.

[44] Toft KG, Hustvedt SO, Hals PA, Oulie I, Uran S, Landmark K, Normann PT, Skotland T (2006). Disposition of perfluorobutane in rats after intravenous injection of Sonazoid. Ultrasound Med Biol.

[45] Dearman RJ, Caddick H, Basketter DA, Kimber I (2000). Divergent antibody isotype responses induced in mice by systemic exposure to proteins: a comparison of ovalbumin with bovine serum albumin. Food Chem Toxicol.

[46] Lei HY, Lee SH, Leir SH (1996). Antigen-induced anaphylactic death in mice. Int Arch Allergy Immunol.

[47] Bhutani MS, Gress FG, Giovannini M, Erickson RA, Catalano MF, Chak A, Deprez PH, Faigel DO, Nguyen CC (2004). No Endosonographic Detection of Tumor (NEST) Study. The No Endosonographic Detection of Tumor (NEST) Study: a case series of pancreatic cancers missed on endoscopic ultrasonography. Endoscopy.

[48] Ueda K, Yamashita Y, Itonaga M (2013). Real-time contrast-enhanced endoscopic ultrasonography-guided fine-needle aspiration (with video). Dig Endosc.

[49] Whiting PF, Rutjes AW, Westwood ME, Mallett S, Deeks JJ, Reitsma JB, Leeflang MM, Sterne JA, Bossuyt PM (2011). QUADAS-2 Group. QUADAS-2: a revised tool for the quality assessment of diagnostic accuracy studies. Ann Intern Med.

